# Structural Basis for Species Specific Inhibition of 17β-Hydroxysteroid Dehydrogenase Type 1 (17β-HSD1): Computational Study and Biological Validation

**DOI:** 10.1371/journal.pone.0022990

**Published:** 2011-08-09

**Authors:** Tobias Klein, Claudia Henn, Matthias Negri, Martin Frotscher

**Affiliations:** 1 Pharmaceutical and Medicinal Chemistry, Saarland University, Saarbrücken, Germany; 2 Department of Drug Design and Optimization, Helmholtz Institute for Pharmaceutical Research Saarland (HIPS), Saarbrücken, Germany; University of Queensland, Australia

## Abstract

17β-Hydroxysteroid dehydrogenase type 1 (17β-HSD1) catalyzes the reduction of estrone to estradiol, which is the most potent estrogen in humans. Inhibition of 17β-HSD1 and thereby reducing the intracellular estradiol concentration is thus a promising approach for the treatment of estrogen dependent diseases. In the past, several steroidal and non-steroidal inhibitors of 17β-HSD1 have been described but so far there is no cocrystal structure of the latter in complex with 17β-HSD1. However, a distinct knowledge of active site topologies and protein-ligand interactions is a prerequisite for structure-based drug design and optimization. An elegant strategy to enhance this knowledge is to compare inhibition values obtained for one compound toward ortholog proteins from various species, which are highly conserved in sequence and differ only in few residues. In this study the inhibitory potencies of selected members of different non-steroidal inhibitor classes toward marmoset 17β-HSD1 were determined and the data were compared with the values obtained for the human enzyme. A species specific inhibition profile was observed in the class of the (hydroxyphenyl)naphthols. Using a combination of computational methods, including homology modelling, molecular docking, MD simulation, and binding energy calculation, a reasonable model of the three-dimensional structure of marmoset 17β-HSD1 was developed and inhibition data were rationalized on the structural basis. In marmoset 17β-HSD1, residues 190 to 196 form a small α-helix, which induces conformational changes compared to the human enzyme. The docking poses suggest these conformational changes as determinants for species specificity and energy decomposition analysis highlighted the outstanding role of Asn152 as interaction partner for inhibitor binding. In summary, this strategy of comparing the biological activities of inhibitors toward highly conserved ortholog proteins might be an alternative to laborious x-ray or site-directed mutagenesis experiments in certain cases. Additionally, it facilitates inhibitor design and optimization by offering new information on protein-ligand interactions.

## Introduction

**Figure 1:**
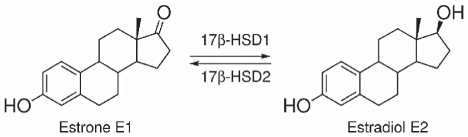
Interconversion of estrone (E1) and estradiol (E2).

Human 17β-hydroxysteroid dehydrogenase type 1 (17β-HSD1) catalyzes the NAD(P)H dependent reduction of the weak estrogen estrone (E1) to the biologically most active estrogen estradiol (E2; Fig. 1) [1]. This reaction, which represents the last step in E2 biosynthesis, takes place in target cells where the estrogens exert their effects via the estrogen receptors α and β. Besides their physiological effects, estrogens are involved in the development and the progression of estrogen dependent diseases (EDDs) like breast cancer, endometriosis and endometrial hyperplasia [2]–[4]. In the past few years, aromatase inhibitors have been intensively investigated for the treatment of EDDs [5]–[7] but they lead to unwanted side effects due to their strong reduction of estrogen levels in the whole body. Therefore reducing local E2 levels by inhibition of 17β-HSD1 is a promising therapeutic approach for the treatment of EDDs. An analogous intracrine concept has already been proved successful for the treatment of androgen dependent diseases such as benign prostatic hyperplasia and alopecia by using 5α-reductase inhibitors [8]–[11]. 17β-HSD2 catalyzes the reverse reaction (oxidation of E2 to E1; Fig. 1) and inhibition of this enzyme must be avoided for the therapeutic concept to work. However, specific inhibition of 17β-HSD2 in bone cells may provide a novel approach to prevent osteoporosis [12].

17β-HSD1 is a cytosolic enzyme that belongs to the superfamiliy of short-chain dehydrogenases/reductases (SDRs) [13]. It consists of 327 amino acid residues (34.9 kDa) and the active form exists as homodimer [14]. 17β-HSD1 comprises a Rossmann fold, associated with cofactor binding, and a steroid-binding cleft [15]. The latter is described as a hydrophobic tunnel with polar residues at each end: His221/Glu282 on the C-terminal side, and Ser142/Tyr155, belonging to the catalytic tetrad, which is present in the majority of characterized SDRs [16], on the other side [17]. To date 22 crystal structures of 17β-HSD1 are available as apoform, binary or ternary complexes [18]–[20]. All crystal structures show an overall identical tertiary structure, while major differences have been identified only for the highly flexible βFαG'-loop. It is not resolved in ten crystal structures, while the remaining twelve showed high b-factor values for this area, which is an additional hint for the flexibility of the βFαG'-loop. In some crystal structures a short α-helix was observed in the loop region but its occurrence seems not to be dependent on the presence of steroidal ligands, cofactor or inhibitor. However, the position and length of the α-helix changes: in the apoform (PDB entry 1bhs) the helix is limited to the beginning of the loop while in presence of steroidal ligands and/or cofactor it is shifted to the end (PDB entries 1dht, 1equ, and 1iol). Further, dependent on the presence of cofactor and ligands, the βFαG'-loop can occupy three possible orientations: an opened, semi-opened, and closed enzyme conformation [21].

Several steroidal and non-steroidal inhibitors of 17β-HSD1 have been described [18], [22]–[37], but only for the former cocrystal structures exist. While several computational studies have been performed in order to elucidate the interactions of non-steroidal inhibitors with 17β-HSD1 [26], [27], [33], [37], [38], structural data confirming the results are still missing.

However, a distinct knowledge of active site topologies and protein-ligand interactions is a prerequisite for structure-based drug design and optimization. To further increase this knowledge, inhibition values obtained for one compound toward proteins, differing only in few residues might be advantageous. For this purpose, wild type proteins and their mutants carrying a set of point mutations can be used. As alternative, proteins from various species, which are highly conserved in sequence and differ only in few residues, might be considered.

This latter approach was applied in the present study employing human and marmoset monkey (*callithrix jacchus*) 17β-HSD1. Selected human 17β-HSD1 inhibitors, representative of our structurally diverse inhibitor classes, were tested toward the marmoset 17β-HSD1. The resulting inhibitory potencies were compared with those obtained for the human enzyme and remarkable differences were only observed in the class of the (hydroxyphenyl)naphthols. In order to rationalize the species-specific inhibition profiles at the structural basis, a homology model of the marmoset enzyme was built using a human 17β-HSD1 x-ray structure as template. Further, the docking poses of selected compounds into both the human crystal structure and the modelled marmoset 17β-HSD1 were considered. Notably, the marmoset homology model and the docking poses of the inhibitors presented herein were validated by their ability to explain inhibition data. Subsequently, the complexes of two representative inhibitors, docked into the marmoset model and the human crystal structure, respectively, were subjected to MD simulations to investigate their conformational equilibrium. In addition, binding energy calculations as well as energy decomposition analysis were performed, with the aim to investigate the influence of the marmoset amino acid variations on the inhibitory potencies. The current work provides new insights into the marmoset 17β-HSD1 active site topology, reveals probable inhibitor binding modes in human and marmoset 17β-HSD1, and identifies amino acids responsible for species specificity in 17β-HSD1 inhibition.

## Results

### Comparison of 17β-HSD type 1 and type 2 sequences

**Figure 2:**
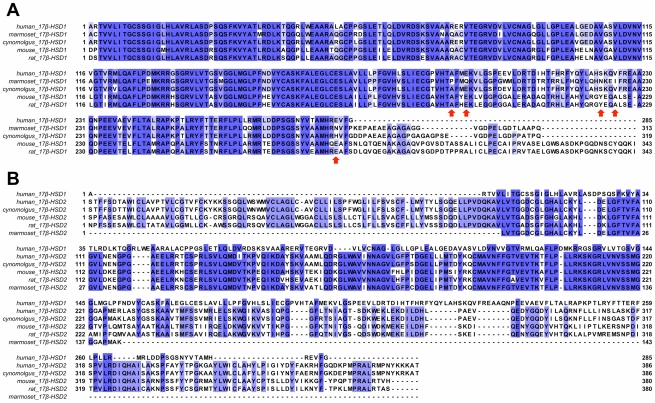
Multiple sequence alignment of 17β-HSD type 1 and type 2 from selected species. Species and subtype are indicated on the left, followed by residue numbering. Residues are colored by percentage identity and red arrows indicate major amino acid variations in marmoset compared to the other species. **A**) Multiple sequence alignment of 17β-HSD1 from selected species. **B**) Multiple sequence alignment of human 17β-HSD1 with 17β-HSD2 from selected species.

To identify regions that are conserved through several species, the sequences of rodent, cynomolgus, marmoset and human 17β-HSDs 1 were aligned (Fig. 2A). The N-terminal region (residues 1–190), which constitutes the common Rossmann fold as well as the catalytic tetrad, is highly conserved for the analyzed species. Remarkable differences were observed in the F/G segment (residues 191–230), which is lining the steroid-binding site, and the C-terminal region (residues 231–285). Sequence alignment of human and marmoset 17β-HSD1 revealed that they share 80% sequence identity and 85% similarity (Fig. 2A). Focusing on the steroid-binding site (residues 94 to 196 and 214 to 284), the identity increases to 87%, with five major amino acid variations observed in the marmoset enzyme: A191P, E194Q, S222N, V225I and E282N. In contrast, comparing cynomolgus and human 17β-HSD1, which show even 91% identity, only one of the aforementioned amino acid variations can be found (E282H; Fig. 2A). With the exception of marmoset 17β-HSD1, Ala191 and Glu194 are conserved through the analyzed species (Fig. 2A) indicating the significance of the observed variations between human and marmoset. 17β-HSDs 1 of mouse and rat are 83% similar to the human enzyme in the first 287 amino acids. In both analyzed rodent enzymes His221, which is involved in steroid binding [17], is mutated into a glycine (H221G). Moreover, several other amino acids of the substrate-binding site are replaced by more bulky residues (L96F, N152H, M193Y/H, S222Y). Interestingly, rat and mouse 17β-HSDs 1 are significantly less sensitive to inhibition by steroidal inhibitors compared to the human ortholog [39] and different classes of non-steroidal potent human 17β-HSD1 inhibitors turned out to be only weak inhibitors of E2 formation in rat liver preparations [40]. This might be partially explained by the absence of His221 as interaction partner as well as by the reduced volume of the active site (M193Y/H, S222Y).

Sequence identities between human 17β-HSD2 and 17β-HSD2 of the selected species range from 61% (mouse) to 93% (cynomolgus) while the F/G segment and the C-terminal region show the most pronounced variability (Fig. 2B). The comparison of 17β-HSD1 with the correspondent type 2 enzymes of the analyzed species revealed, that the type 2 enzymes have about 80 additional N-terminal residues relative to the type 1 enzymes. Sequence comparison showed that they share a very low overall sequence identity (≤25%) with major differences in the F/G segment and the C-terminal domain, which constitute the active site. However, some amino acid motifs, characteristic for SDR enzymes [18], are highly conserved: the T-G-xxx-G-x-G motif, the Y-xxx-K sequence and the N-A-G motif (Fig. 2B). For marmoset 17β-HSD2 only a fragment of the primary sequence is available, which is 143 amino acids in length and constitutes the Rossman fold whereas the F/G segment and the C-terminal region are missing (Fig. 2B). This segment is 29% identical to marmoset 17β-HSD1.

As human and marmoset 17β-HSD1 differ in few residues of the active site, comparison of the inhibitory potencies of selected inhibitors, observed toward both enzymes, is suitable to further increase the knowledge of active site topologies and protein-ligand interactions. In particular, the amino acid variations in the flexible βFαG'-loop, which is suggested to play a crucial role in ligand binding, will be used to elucidate the function of the loop in more detail.

### Inhibition of marmoset 17β-HSD1 and 17β-HSD2

Marmoset placental tissue was used as enzyme source and the proteins were partially purified following a described procedure [41]. Tritiated E1 was incubated with 17β-HSD1, cofactor and inhibitor. The separation of substrate and product was performed by HPLC. In an assay similar to the 17β-HSD1 test, marmoset placental microsomes containing 17β-HSD2 were incubated with tritiated E2 in the presence of NAD^+^ and inhibitor. Labelled product was quantified after HPLC separation.

**Figure 3:**
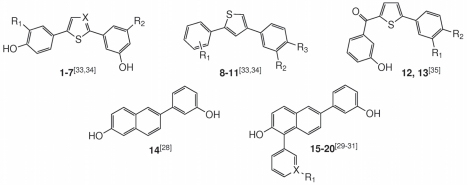
Chemical structures of selected human 17β-HSD1 inhibitors. Representative structures of our three inhibitor classes: the bis(hydroxyphenyl) substituted arenes (**1–11**), the bicyclic substituted hydroxyphenylmethanones (**12**, **13**) and the (hydroxyphenyl)naphthols (**14–20**).

The inhibition values of compounds **1–20** (Fig. 3) are shown in Table 1. The bis(hydroxyphenyl) substituted arenes **1–11** showed comparable or even higher inhibitory potencies toward marmoset 17β-HSD1 compared to the human enzyme. For example, compound **8**, which is a potent inhibitor of human 17β-HSD1 (IC_50_ = 151 nM), showed a stronger inhibitory potency toward marmoset 17β-HSD1 (IC_50_ = 4 nM). Comparable inhibition data for human and marmoset 17β-HSD1 were also measured for the bicyclic substituted hydroxyphenylmethanones **12** and **13**. This indicates that the binding of the two aforementioned inhibitor classes is reinforced or at least not affected by the amino acid variations in marmoset 17β-HSD1. In the class of the (hydroxyphenyl)naphthols, the human 17β-HSD1 inhibitor **14** (IC_50_ = 116 nM) showed also a good inhibitory potency toward marmoset 17β-HSD1 (IC_50_>50 nM). The introduction of space filling substituents in position 1 of the naphthol core of **14** is beneficial for the inhibition of the human enzyme (**16**, IC_50_ = 26 nM), but for marmoset 17β-HSD1 no increase in inhibitory potency was observed (**16**, IC_50_>50 nM) compared to the unsubstituted **14**. Interestingly, further enlargement of the substituents in 1-position of **14** led to highly active human 17β-HSD1 inhibitors (**19**, IC_50_ = 15 nM) while a reduced potency toward marmoset 17β-HSD1 (**19**, IC_50_>50 nM) was found.

**Table 1:** Inhibition of human and marmoset 17β-HSD1 and 17β-HSD2 by compounds 1–20.

					Human IC_50_ [nM]		Marmoset IC_50_ [nM]^c^	
compd	X	R_1_	R_2_	R_3_	17ß-HSD1^a^	17ß-HSD2^b^	17ß-HSD1^d^	17ß-HSD2^e^
**1**	CH	F	H		8	940	<5^f^	>50
**2**	N	H	H		50	4004	102	>50
**3**	CH	H	H		69	1953	31	>50
**4**	CH	CH_3_	H		46	1971	<50	>50
**5**	N	CH_3_	H		143	2023	<50	>50
**6**	CH	H	F		42	463	<5^f^	85
**7**	CH	F	F		17	218	<5^f^	43
**8**		4-OH	OH	H	151	1690	4^f^	>50
**9**		3-OH	H	OH	77	1271	2^f^	>50
**10**		3-OH	CH_3_	OH	64	869	3^f^	>50
**11**		3-OH	F	OH	64	510	<50	72
**12**		H	OH		33	478	<50	43
**13**		OC_2_H_5_			78	502	<50	59
**14**					116	5641	>50	>50
**15**	C	OH			36	959	32	>50
**16**	N				26	1157	>50	>50
**17**	C	H			20	540	52	>50
**18**	C	NH_2_			53	1757	n.i.	>50
**19**	C	NHSO_2_CH_3_			15	403	>50	>50
**20**	C	NHCOCH_3_			83	1239	>50	n.i.

a Human placental, cytosolic fraction, substrate E1, 500 nM, cofactor NADH, 500 µM;

b Human placental, microsomal fraction, substrate E2, 500 nM, cofactor NAD^+^, 1500 µM;

c Logit transformed values calculated from % inhibition at 50 nM inhibitor concentration, for inhibition values <30% or >70%, a trend is given;

d Marmoset monkey placental, cytosolic fraction, substrate E1, 500 nM, cofactor NADH, 500 µM;

e Marmoset monkey placental, microsomal fraction, substrate E2, 500 nM, cofactor NAD^+^, 1500 µM;

f Inhibitor concentration: 5 nM; n.i.: no inhibition; Human IC_50_ values were retrieved from literature (corresponding references are indicated with the structural formulas in Fig. 3).

The inhibitory potencies of compounds **1–20** toward marmoset 17β-HSD2 were also determined to prove whether marmoset monkey is a suitable species for *in vivo* evaluation of 17β-HSD1 inhibitors (Table 1). Remarkably, lower selectivity of compounds toward non-target marmoset 17β-HSD2 was observed, when comparing to human 17β-HSD2.

### Homology modelling and MD simulations of marmoset 17β-HSD1

**Figure 4:**
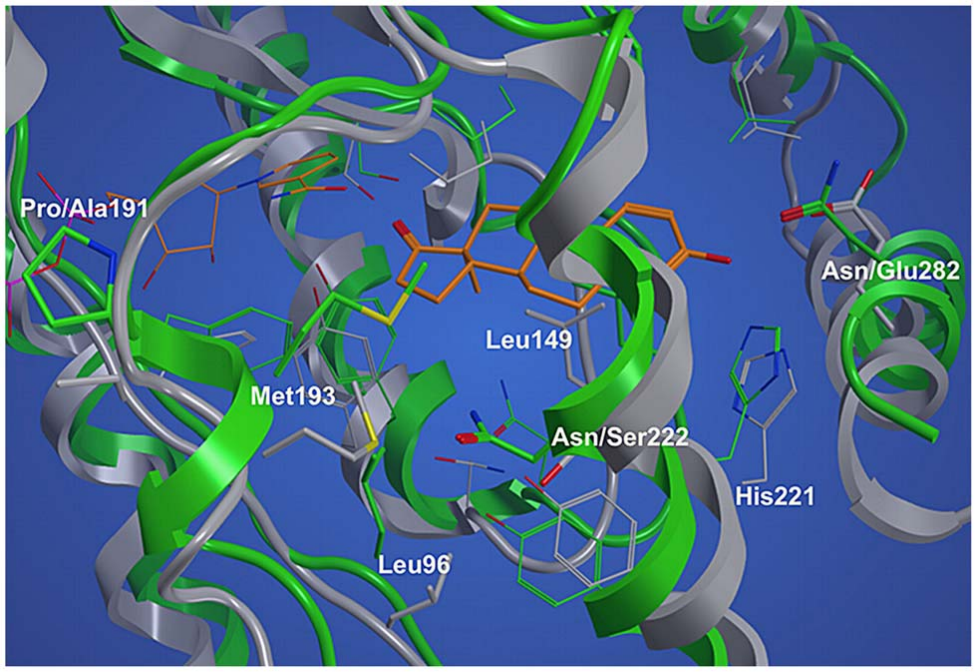
Three-dimensional structure superimposition of marmoset and human 17β-HSD1. Low energy structure of marmoset 17β-HSD1 (green) in complex with E1 and NADPH (orange) from MD simulation superimposed onto the x-ray structure of human 17β-HSD1 (grey, PDB code: 1fdtB). When two residues are indicated, the first corresponds to marmoset 17β-HSD1 and the second to human 17β-HSD1.

In order to obtain a more precise picture of the three-dimensional structure of the marmoset 17β-HSD1, a homology model was generated. A set of 100 models was built with MODELLER 9v7 [42] using the ternary complex E1-NADPH-human 17β-HSD1 as template. This complex was obtained by docking E1 into human 17β-HSD1 employing the Protein Data Bank (PDB) entry 1fdt with conformation B for residues 187–200 (in the following determined as 1fdtB). This 3D structure was chosen as it represents a ternary complex with NADP^+^ and E2 in the closed enzyme conformation, as the βFαG'-loop is resolved, and as this protein structure was already successfully used in previous docking studies with the investigated compound classes [30], [36]. The best model was chosen according to internal DOPE-score [43] and PROCHECK [44] tests. Notably, it presents a short α-helix (residues 190–196) in the region between the βF-sheet (residues 178–186) and the αG'-helix (residues 209–227), with Pro191 in the first turn of the helix (N1 position; Fig. 4), thus differing from the secondary structure of the template.

**Figure 5:**
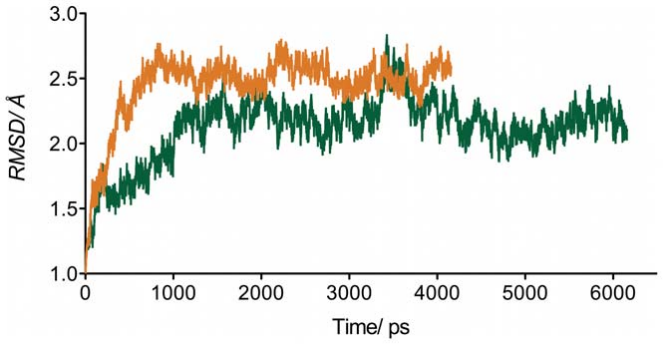
RMSD analysis of the MD simulations of the marmoset 17β-HSD1 model. Time-dependent C_α_-RMSD for all residues of the secondary (orange) and ternary (green) complex.

The selected model was further refined both as holoenzyme with NADPH and as ternary complex with NADPH and E1 by MD simulations. The trajectories of the two MD simulations were stable with a α-carbon root-mean-square deviation (RMSD) to the starting structure below 3.0 Å (Fig. 5). No major structural differences were observed when comparing the simulated holoenzyme and the ternary complex. Therefore, the following results, based on analysis of the ternary complex, also apply to the holoform.

After an initial inward rotation in the MD simulation, the short α-helix (residues 190–196) remained stable for the last 5 ns of the MD simulation, with an average backbone RMSD to the final conformation of 1.03 Å. Remarkably, a similar behaviour cannot be expected for the region between the βF-sheet and the αG'-helix in the human enzyme. The differences in the crystal structures suggest a high flexibility for the βFαG'-loop: it is not resolved in ten crystal structures and the remaining twelve show high b-factor values for this area. Some crystal structures present a short α-helix in the loop region whose position and length varies dependent on the presence of steroidal ligands, cofactor or inhibitor. In the apoform (PDB entry 1bhs) the helix is limited to the beginning of the loop, whereas in presence of steroidal ligands and/or cofactor it is shifted to the end (PDB entries 1dht, 1equ, and 1iol). Further, in the human enzyme, the βFαG'-loop axis occupies different orientations dependent on the presence of cofactor and ligands. In the marmoset however, both the holoform and the ternary complex show a helix starting already at the beginning of the βFαG'-loop with its axis in only one conformation.

The presence of the newly formed α-helix (residues 190–196) induced a different orientation of the side chain of Met193. Compared to the template structure, Met193 protrudes deeper into the substrate-binding site and stabilizes E1 by hydrophobic interactions (Fig. 4). Furthermore, during MD simulation a kink in the loop between the βD-sheet and the αE-helix was observed. Thereby the side chain of Leu96 was brought closer to E1 allowing Van der Waals contacts (Fig. 4). Summarizing, in the final part of the MD simulation E1 was stabilized by both lipophilic interactions and hydrogen bonds: Leu96, Leu149, Met193, and Phe259 constrained the steroidal scaffold while Ser142/Tyr155 and His221/Asn282 interacted with the carbonyl oxygen in 17-position and the 3 OH-group, respectively. The latter residue took over the H-bond acceptor abilities of Glu282, which is involved in forming an H-bond with the 3 OH-group of E1 in the human enzyme.

Employing CASTp [45], the active site volumes of human and marmoset 17β-HSD1 were calculated. The above-described conformational changes of Leu96 and Met193 as well as the S222N and V225I mutations resulted in a reduced volume of the marmoset 17β-HSD1 active site (478 Å^3^) compared to that of the human ortholog (627 Å^3^).

The stereochemical quality of the holoenzyme and the ternary complex models obtained from MD simulations was checked with PROCHECK [44]. The majority of the residues of the investigated structures were found to occupy the most favoured regions of the Ramachandran plots, while the other residues occupied the additional allowed regions. In detail, in the ternary complex 78.1% of the residues were placed in the most favoured region, 20.2% in the additional allowed region, 1.2% in the generously allowed region, and only 0.4% in the disallowed region (for the holoform: 81.8%, 17.4%, 0.8% and 0%).

### Molecular docking

**Figure 6:**
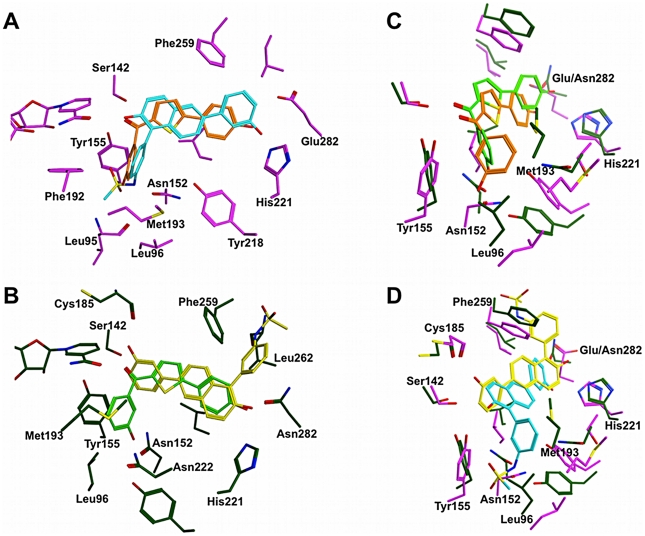
Hypothetical binding modes of compounds 12 and 19. **A**) Superimposition of lowest energy structures of **12** (orange) and **19** (cyan) obtained by docking into the x-ray structure of human 17β-HSD1 (magenta, PDB code: 1fdtB). **B**) Superimposition of lowest energy structures of **12** (light green) and **19** (yellow) obtained by docking into the marmoset 17β-HSD1 homology model (green). **C**) Superimposition of lowest energy structures of **12** (light green) docked into the marmoset 17β-HSD1 model (green) and of **12** (orange) docked into the x-ray structure of human 17β-HSD1 (magenta, PDB code: 1fdtB). **D**) Superimposition of lowest energy structures of **19** (yellow) docked into the marmoset 17β-HSD1 model (green) and of **19** (cyan) docked into the x-ray structure of human 17β-HSD1 (magenta, PDB code: 1fdtB).

As the potent inhibitors of human 17β-HSD1 **12** and **19** are structurally diverse and exhibit different potencies toward marmoset 17β-HSD1 they were chosen as representatives to rationalize the observed species-specific inhibition profiles. Employing AutoDock 4 [46], both compounds were docked into the active sites of marmoset and human 17β-HSD1 using the equilibrated, ternary complex after 5537 ps and the x-ray structure 1fdtB (see experimental part), respectively. The inhibitor poses used for further investigation were selected considering binding energy and statistical representativity (cluster population; Table S1) and are shown in Figure 6.

In the human enzyme both inhibitors are placed in the substrate-binding site and they occupy an apolar subpocket consisting of the following amino acids: Gly94, Leu95, Leu96, Asn152, Tyr155, and Phe192 (Fig. 6A). While the carbonyl group of compound **12** mimics the D-ring keto function of E1, forming H-bonds with Ser142 and Tyr155, its *para*-OH group resembles the 3-OH of E1, which interacts with His221 via an H-bond. The *meta*-hydroxyphenyl moiety is projecting into the subpocket, where it forms an additional H-bond with Asn152 and is stabilized by π-π-interactions with Tyr155 and Phe192.

The (hydroxyphenyl)naphthol-core of compound **19** occupies the substrate-binding site and is stabilized by three H-bonds: the 2-OH group interacts with Ser142 as well as with Tyr155 and the OH group in *meta* position of the phenyl ring in 6-position interacts with His221. In this case, the sulfonamide substituted phenyl ring in 1-position of the naphthol core protrudes into the subpocket, where it is stabilized by H-bonds with Asn152 and with the -NH- of the backbone of Leu95 (Fig. 6A).

Also in the marmoset enzyme both compounds occupy the substrate-binding site, but only **12** protrudes into the apolar subpocket (Fig. 6B). Due to the altered side chain conformation of Leu96 in the marmoset enzyme, compound **12** is slightly displaced toward the C-terminus (Fig. 6C). Regarding the interaction pattern, only minor changes were observed: the carbonyl group forms only an H-bond with Ser142 but for the *para*-OH group a second H-bond with Asn282 was observed (Fig. 6C).

Interestingly, compound **19** resulted in a completely different binding mode when docked into the homology model of marmoset 17β-HSD1 with respect to its position in the human crystal structure 1fdtB (Fig. 6D). The OH-group in *meta* position of the phenyl ring makes an H-bond with the backbone carbonyl oxygen of Cys185 and the 2-OH function forms H-bonds with His221 and Asn282 in a bifurcated fashion. The sulfonamide substituted phenyl ring is located in the C-terminal gate and might be stabilized by π-π-interactions with Phe259 and an H-bond with the backbone -NH- of Leu262.

### Validation of the docking complexes by means of MD simulations and free energy calculations (MM/PBSA)

**Figure 7:**
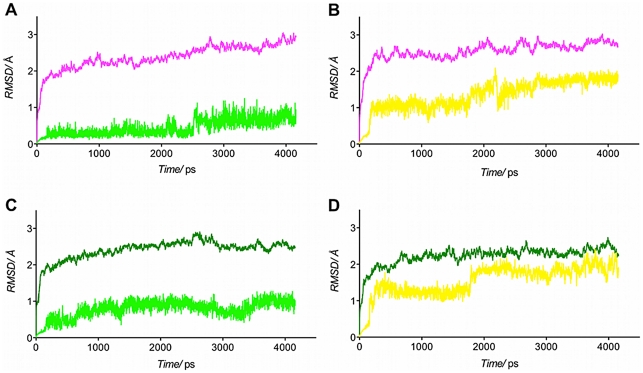
Time dependent RMSD analysis of Cα of 17β-HSD1 and of the heavy atoms in the ligands. **A**) C_α_-RMSD of human 17β-HSD1 is colored in magenta and the heavy atoms RMSD of compound **12** in light green. **B**) C_α_-RMSD of human 17β-HSD1 is colored in magenta and the heavy atoms RMSD of compound **19** in yellow. **C**) C_α_-RMSD of marmoset 17β-HSD1 is colored in green and the heavy atoms RMSD of compound **12** in light green. **D**) C_α_-RMSD of marmoset 17β-HSD1 is colored green and the heavy atoms RMSD of compound **19** in yellow.

With the aim to validate the docking results and to unravel possible induced-fit mechanisms, different MD simulations were run in explicit aqueous solution. Distance restraints were applied to inhibitors only in the first ns of the MD simulations with the aim of maintaining their proper orientation. For the rest of the MD simulation no restraints were used and the whole complexes were left free to move. This was done in order to avoid trapping the inhibitor in an unstable conformation, which could bias the results. The RMSD values of the heavy atoms of the inhibitors and of the Cαatoms of the enzymes were analyzed as a function of time to assess the degree of conformational drift, as shown in Figure 7.

In the simulation of **12** bound to human 17β-HSD1 the CαRMSD of the protein as well as the RMSD of the heavy atoms of **12** showed a stable plateau (∼2.2 Å) from 1.0 to 2.5 ns (Fig. 7A). After 2.5 ns the CαRMSD of the protein increased and a minor fluctuation of the heavy atom RMSD of **12** was observed. The latter finding could be related to a slight shift of the inhibitor toward the C-terminal end of the enzyme. Notably, the hydrogen bonds between **12** and 17β-HSD1, which were observed in the initial structure, were conserved during the 4 ns simulation suggesting that both protein and **12** fluctuations do not impact the inhibitor binding.

Both human 17β-HSD1 and compound **19** were stable in the simulation of their complex (Fig. 7B). During the simulation (after 1.5 ns) the hydrogen bond of the *meta*-OH group with His221 is replaced by an H-bond with Glu282. However, after 2.1 ns, this hydrogen bond is interrupted for 0.2 ns allowing the hydroxyphenyl ring to rotate freely around the axis of the bond to the naphthol core thereby inducing a minor fluctuation of the heavy atom RMSD of **19**. After this short interruption the H-bond interaction with Glu282 is re-established. On the other side, the hydrogen bonds between the 2-OH group and Ser142/Tyr155 as well as between the sulfonamide moiety and Asn152/Leu95 endured constant.

During the MD simulation of **12** in complex with marmoset 17β-HSD1, the *meta*-hydroxyphenyl moiety of **12** moved further out of the subpocket. This was reflected by the minor fluctuation of the RMSD of the heavy atoms of **12** after 1.4 ns (Fig. 7C). While this motion caused the break of the H-bond with Asn152, it also placed the *meta*-OH group in an appropriate distance to Asn222, thus allowing a new H-bond formation.

The analysis of the MD simulation of the marmoset 17β-HSD1-**19** complex revealed an overall stable CαRMSD of the protein. However, after 1.8 ns, a ∼0.5 Å fluctuation of the heavy atom RMSD of **19** was observed (Fig. 7D) corresponding to the rotation around the axis of the bond between the hydroxyphenyl ring and the naphthol core. Thereby the H-bond with the backbone amino group of Leu262 was lost.

Furthermore, for each of the four MD trajectories absolute free energy (ΔG) and relative binding affinity (ΔG_bind_) were calculated applying MM/PBSA methods and NMODE analysis (Table 2). This was done for the following stable sectors: 1000 to 2500 ps for **12** in complex with human 17β-HSD1 (Fig. 7A), 2300 to 4160 ps for **19** in complex with human 17β-HSD1 (Fig. 7B), 1500 to 4160 ps for **12** in complex with marmoset 17β-HSD1 (Fig. 7C) and 1800 to 4160 ps for **19** in complex with marmoset 17β-HSD1 (Fig. 7D). All four complexes showed favourable ΔG values, ranging from −7.3 kcal mol^−1^ to −3.2 kcal mol^−1^. The free energies observed for compound **12** in complex with human (ΔG = −4.7 kcal mol^−1^) and marmoset 17β-HSD1 (ΔG = −5.8 kcal mol^−1^) are in the same range. This is in accordance with the inhibitory activities of **12**, which are comparable for both species (see Table 1). Regarding compound **19**, the complex with human 17β-HSD1 shows a more favourable free energy (ΔG = −7.3 kcal mol^−1^) than the one with marmoset 17β-HSD1 (ΔG = −3.2 kcal mol^−1^). This is mainly due to poor entropic contributions in the latter case. Remarkably, this finding is in concert with the experimentally determined inhibition data: compound **19** is a highly potent human 17β-HSD1 inhibitor (IC_50_ = 15 nM) with reduced activity toward the marmoset enzyme (IC_50_>50 nM).

**Table 2:** Free energy calculations for the MD simulations of the four docking complexes.

comp	ELEC	VDW	GAS	PBSOL	PBTOT (ΔG_bind_)	TSTOT	ΔG
		mean	mean	mean	mean (±SE)	mean (±SE)	mean (±SE)
**Human x-ray structure (1fdtB)**							
**12**	−15.4	−40.4	−55.7	35.1	−20.6±3.8	−15.9±6.6	−4.7±7.6
**19**	−36.7	−50.1	−86.8	64.2	−22.6±5.0	−15.3±6.8	−7.3±8.4
**Marmoset monkey homology model**							
**12**	−24.5	−38.1	−61.3	40.0	−21.3±4.9	−15.4±5.3	−5.8±7.2
**19**	−42.2	−34.4	−76.5	50.3	−26.3±4.4	−23.1±4.3	−3.2±6.1

**ΔG** and **ΔG_bind_** values correspond to the longest stable plateau for each MD. (**ΔG**) free binding energy; (**PBTOT**) (**ΔG_bind_**) relative binding energy; (**ELEC**) electrostatic contribution in gas phase; (**VDW**) Van der Waals contribution in gas phase; (**GAS**) free energy in vacuum; (**PBSOL**) solvation energy; (**TSTOT**) (**TΔS**) entropic contribution; (**mean**) mean value; (**SE**) standard error of the mean; all energies expressed in kcal mol^−1^.

### Analysis of the binding interactions using MM/GBSA methods

Focusing on the specific interactions, which mediate the binding of **12** and **19** to human and marmoset 17β-HSD1, we have analyzed the interaction energies of both inhibitors with the residues of the binding sites, employing a pairwise per-residue energy decomposition analysis.

Inspection of the interaction energies with human 17β-HSD1 (Table 3) showed that the hydrogen bonds of **12** (−4.1 kcal mol^−1^) and **19** (−5.7 kcal mol^−1^) with Asn152 contributed most to the interaction energies. Besides Asn152, further residues of the subpocket (Gly94, Leu95, Leu96) interact with both inhibitors revealing energies from −0.3 kcal mol^−1^ to −1.8 kcal mol^−1^. The energies of the hydrogen bonds between **12** and the catalytic residues Ser142 as well as Tyr155 are −1.3 kcal mol^−1^ and −1.5 kcal mol^−1^, respectively. In case of compound **19** the interaction energies are −1.3 kcal mol^−1^ for the H-bond with Ser142 and −1.3 kcal mol^−1^ for the H-bond with Tyr155. Further binding site residues, which significantly contribute to the binding of compounds **12** and **19** (Leu149, Pro187, Phe192, Met193, Val225, and Phe259) show interaction energies from −0.9 kcal mol^−1^ to −2.2 kcal mol^−1^. Regarding the polar amino acids at the C-terminal end of the binding site, His221 takes primarily part in the binding of **12** (−1.8 kcal mol^−1^ for **12** vs. −0.2 kcal mol^−1^ for **19**) while Glu282 is mainly involved in binding compound **19** (−0.1 kcal mol^−1^ for **12** vs. −4.3 kcal mol^−1^ for **19**).

**Table 3:** Interaction energies between the inhibitors 12 and 19 and the proximal (4.0 Å) binding site residues of human 17β-HSD1.

comp	Gly94	Leu95	Leu96	Ser142	Leu149	Asn152	Tyr155	Cys185	Gly186	Pro187
**12**	−1.8	−0.3	−1.2	−1.3	−2.2	−4.1	−1.5	−0.2	−0.4	−1.3
**19**	−0.7	−0.7	−1.7	−1.3	−2.0	−5.7	−2.6	−0.3	−0.8	−2.0

comp	Phe192	Met193	Tyr218	His221	Ser222	Val225	Arg258	Phe259	Leu262	Glu282
**12**	−1.2	−1.0	−0.7	−1.8	−0.8	−1.7	−0.1	−1.2	−0.4	−0.1
**19**	−1.8	−1.9	−0.1	−0.2	−0.4	−1.9	−0.5	−0.9	−0.3	−4.3

All energies are expressed in kcal mol^−1^.

The interaction energies of **12** and **19** with marmoset 17β-HSD1 are listed in Table 4. Remarkably, for inhibitor **12**, the energy contribution of the H-bond with the marmoset Asn152 (−1.6 kcal mol^−1^) is 2.6 fold reduced compared to the human enzyme, while for compound **19** it is almost lost (−0.2 kcal mol^−1^). Both for **12** and **19** reduced energies were also observed for interactions with Gly94, Leu95, and Leu96. Interestingly, inhibitor **12** showed an interaction with Asn222 in the marmoset enzyme (−3.3 kcal mol^−1^), which was not observed for Ser222 in human 17β-HSD1 (−0.8 kcal mol^−1^). This is not the case for **19**, as the interaction with Asn222 (−0.3 kcal mol^−1^) does not contribute significantly to the interaction energy. In addition to Leu149, Pro187, Met193, Ile225, and Phe259, which interact with both compounds in the human enzyme, Gly186, His221, and Asn282 significantly contribute to the binding of **12** and **19** to marmoset 17β-HSD1 with interaction energies in the range from −0.4 kcal mol^−1^ to −3.6 kcal mol^−1^. Interestingly, in marmoset 17β-HSD1 the interaction energy between **12** and Asn282 (−3.2 kcal mol^−1^) is 32 fold increased compared to that with Glu282 in the human enzyme (−0.1 kcal mol^−1^). In marmoset 17β-HSD1 an increased energy is also observed for the interaction of compound **19** with His221 (−2.6 kcal mol^−1^) compared to the human enzyme (−0.2 kcal mol^−1^).

**Table 4:** Interaction energies between the inhibitors 12 and 19 and the proximal (4.0 Å) binding site residues of marmoset monkey 17β-HSD1.

comp	Gly94	Leu95	Leu96	Ser142	Leu149	Asn152	Tyr155	Cys185	Gly186	Pro187
**12**	−0.0	−0.1	−0.8	−2.1	−2.0	−1.6	−0.8	−0.3	−0.7	−2.0
**19**	−0.0	−0.1	−0.2	−0.9	−1.1	−0.2	−0.3	−3.5	−2.0	−2.0

comp	Phe192	Met193	Tyr218	His221	Asn222	Ile225	Arg258	Phe259	Leu262	Asn282
**12**	−0.4	−1.3	−1.4	−0.8	−3.3	−1.9	−0.1	−1.3	−0.9	−3.2
**19**	−0.0	−0.4	−0.0	−2.6	−0.3	−2.6	−3.7	−1.9	−1.0	−3.6

All energies are expressed in kcal mol^−1^.

## Discussion

When the three-dimensional structures of marmoset and human 17β-HSD1 are compared, one of the most striking features is the small α-helix including the residues 190 to 196. It is formed in the segment between the βF-sheet and the αG'-helix starting from the interface residue Thr190, which is half in and half out of the helix (N-cap position). In contrast to the human enzyme, where this region is highly flexible, as suggested by the different crystal structures, the α-helix stayed stable during the MD simulation in both the holoform and the ternary complex. The observed conformational stability might be explained by the presence of a proline in position 191 instead of an alanine. Proline is a favourable candidate for N1 position because of its own conformational properties: with only one rotatable angle it loses less entropy than other amino acids in forming an α-helix and thereby it should have some stabilizing influence [47]. Furthermore, in an analysis of sequence-structural characteristics in protein crystal structures, proline was found to be a favoured residue at N1 position. Especially the residue pair involving threonine at N-cap and proline at N1 position, which is observed for marmoset 17β-HSD1, has a high prevalence [48].

In order to analyse the influence of the conformational changes in marmoset 17β-HSD1 on ligand binding, docking studies with subsequent MD simulations, free energy calculations, and energy decomposition analyses were carried out. While the conformational differences between the marmoset and the human enzyme did not affect the binding mode of **12** remarkably, the suggested binding mode of **19** differed strongly in 17β-HSD1 of both species. One possible explanation for that might be the lower sterical demand of **12** compared to **19**. However, the energy contribution of the interaction between **12** and the marmoset Asn152 is reduced, whereas it was outstanding in complex with the human enzyme. Due to the minimal shift of **12** in the marmoset binding pocket, the geometric parameters for the H-bond with Asn152 are no longer optimal. Interestingly, in the marmoset enzyme an additional interaction of **12** with Asn222 is observed, which seems to compensate the deficit in interaction energy due to the absent interaction with Asn152 resulting in comparable binding energies for **12** in complex with human and marmoset 17β-HSD1. The latter finding is in accordance with the inhibition data observed for compound **12** and validates the marmoset 17β-HSD1 model.

Considering compound **19**, no particular interactions with the subpocket residues of the marmoset enzyme exist. Although weak interactions between **19** and the C-terminal region of marmoset 17β-HSD1 are observed, the binding free energy is less favourable compared to that calculated for the human 17β-HSD1-**19** complex. As the C-terminal part of the enzyme has already been discussed as a potential product exit gate of the enzyme [21], inhibitor **19** might be solvent exposed. This is consistent with the unfavourable entropy term of this complex resulting in the least favourable free energy.

Obviously, the presence of a proline in the flexible loop region and the thereby induced conformational changes in marmoset 17β-HSD1 are decisive for the species specific inhibition of **19**. On one hand interactions with subpocket residues like Asn152, recently discussed as relevant interaction partner [49], are prevented and on the other hand the inhibitor is forced in an unfavourable solvent exposed conformation.

The bis(hydroxyphenyl) substituted arenes (compounds **1–11**) show similar or increased inhibitory potencies toward marmoset 17β-HSD1 when comparing to human 17β-HSD1. Recently performed docking experiments proposed a steroidal binding mode when the human crystal structure 1fdtB was used [33]. The high inhibitory potencies toward marmoset 17β-HSD1 are in concert with the modelled structure of marmoset 17β-HSD1 as steroid-like binding is not affected by the proposed conformational changes. Obviously, they even stabilize the bis(hydroxyphenyl) substituted arenes in the marmoset 17β-HSD1 binding pocket as indicated by the observed inhibitory potencies.

Differing inhibitory potencies toward human 17β-HSD1 and 17β-HSD2 may arise from sequence variations in the regions 94–196 and 214–284 (numbering according to 17β-HSD1), which might lead to differences in the active sites of the two human subtypes. A lower selectivity of compounds toward non-target marmoset 17β-HSD2 was observed, when comparing to human 17β-HSD2. Obviously, the differences in the active sites of marmoset 17β-HSD1 and 17β-HSD2 are less pronounced compared to the human orthologs. However, as the available marmoset 17β-HSD2 sequence is missing the F/G segment and the C-terminal part this hypothesis cannot be proved.

The validity of the presented homology model is further substantiated by its ability to explain the reduced inhibitory potency of C-15 substituted estrone derivatives toward marmoset 17β-HSD1 [39]. The substituents in 15-position of the steroid were designed to occupy the hole between the flexible βFαG'-loop and the αG'-helix in the human enzyme [50]. Together with the helix formation and the conformational changes in the βD/αE-segment, the S222N mutation limits the size of the hole in marmoset 17β-HSD1 and thereby might reduce the inhibitory potency toward the marmoset enzyme.

### Conclusion

An elegant strategy to gain more knowledge of active site topologies and, in particular, of protein-ligand interactions is to compare inhibition values obtained for one compound toward ortholog proteins from various species, which are highly conserved in sequence and differ only in few residues. Thereby, such an approach can be a valid alternative to site-directed mutagenesis. As human and marmoset 17β-HSD1 enzymes meet these criteria, selected human 17β-HSD1 inhibitors were assessed for their inhibitory potencies toward marmoset 17β-HSD1. While a species specific inhibition profile was observed in the class of the (hydroxyphenyl)naphthols, representatives of the other evaluated compound classes showed similar or even higher inhibition compared to those observed for the human enzyme. Using a combination of computational methods, including homology modeling, molecular docking, MD simulation, and binding energy calculation, a reasonable model of the three-dimensional structure of marmoset 17β-HSD1 was developed and inhibition data were rationalized on the structural basis. In the marmoset 17β-HSD1, residues 190 to 196 form a small α-helix, which is obviously stabilized by the presence of a proline in N-cap position (residue 191) and induces conformational changes that affect ligand binding. Furthermore energy decomposition analysis highlighted the important role of Asn152 as interaction partner for inhibitor binding.

This work could not only offer a better understanding of the active site topologies and of the protein-ligand interactions, but also provides novel structural clues that will help to design and optimize potent human 17β-HSD1 inhibitors with improved inhibitory potency toward marmoset 17β-HSD1. This is an important step to turn compounds, which show a promising pharmaceutical profile, into candidates for *in vivo* evaluation. Thus, our combined computational approach could also be considered as a valuable tool to achieve this goal.

## Methods

### Sequence Alignment and Model Building

The amino acid sequences of rat (accession number P51657), mouse (P51656) cynomolgus (Q4JK77) and marmoset 17β-HSD1 (Q9GME2) as well as human (P37059), cynomolgus (Q4JK76), marmoset (Q9GME5), mouse (P51658) and rat (Q62730) 17β-HSD2 were obtained from the uniprot webpage. These sequences were pairwise aligned with human 17β-HSD1 (PDB code: 1fdt) using MAFFT version 5 [51]. Using this alignment, a set of 100 comparative models of marmoset 17β-HSD1 was built employing Modeller9v7 [42], with the ternary complex E1-NADPH-human 17β-HSD1 as template. This complex resulted from docking of E1 to human 17β-HSD1 (PDB code: 1fdtB) (see below). The best homology model was then selected according to the Modeller energy score, DOPE score [43] and PROCHECK [44] tests. The reliability of the built homology models was checked by Prosa2003 [52] (Fig. S1), ERRAT [53], and Verify3D [54] (Fig. S2).

### MD Simulations

MD simulations were performed using the AMBER 9.0 suite program [55]. The partial atomic charges for E1 and the inhibitors were derived from the molecular electrostatic potential (MEP) previously calculated using GAMESS [56], according to the RESP methodology [57]. For the protein, partial atomic charges were read from the AMBER 9.0 libraries. The AMBER99SB force field [58] was employed to define atom types and potentials for the protein, while the general AMBER force field (gaff) [59] was used to define all needed atom types and parameters for E1 and the inhibitors. For NADPH (charge −4), the parameters previously reported by Ulf Ryde were applied (http://www.teokem.lu.se/~ulf/).

The input files for the MD simulation were prepared with the xLEaP module of AMBER. Each system was solvated with an octahedral box of TIP3P water molecules of 10 Å radius and neutralized by the addition of Na^+^ ions. Finally, for each complex the topology and the coordinate files were written and used in the MD simulations.

Before starting the production-run phase, the following equilibration protocol was applied to all systems. At the beginning the system was energy-minimized in two stages: firstly, the solvent was relaxed while all the solute atoms were harmonically restrained to their original positions with a force constant of 100 kcal mol^−1^ Å^−2^ for 1000 steps; and secondly, the whole molecular system was minimized for 2500 steps by conjugate gradient. Subsequently, the system was heated during 60 ps from 0 to 300 K at constant volume conditions (NTV, PBC conditions), and then equilibrated keeping both temperature and pressure constant (NTP, PBC conditions, 300 K, 1 atm) during 100 ps. Electrostatic interactions were computed using the Particle Mesh Ewald method [60], and the SHAKE [61] algorithm was employed to keep all bonds involving hydrogen atoms rigid. NADPH, E1 and the inhibitors were constrained during the equilibration with a force constant of 20 kcal mol^−1^ Å^−2^. After equilibration, a MD production stage (NTP, PBC conditions, 300 K, 1 atm) was performed. The total simulation length differed for the various complexes ranging from 4 to 6 ns. Distance restraints were applied to substrate/inhibitor with the aim of maintaining their proper orientation at the beginning (first ns) of production stage.

For the ternary complex of marmoset 17β-HSD1 with E1 and NADPH, two additional distance restraints were used: between the keto-oxygen of E1 and the side chain oxygen of Ser142 (d = 2.40–3.00 Å; force constant: 10 kcal mol^−1^ Å^−2^) and between the oxygen of the OH-group in 17-position of E1 and the NE2 nitrogen of the His221 side chain (d = 2.70–3.40 Å; force constant: 10 kcal mol^−1^ Å^−2^).

For the ternary complex of human 17β-HSD1 with **12** and NADPH, three additional distance restraints were used: between the keto-oxygen of **12** and the side chain oxygen of Tyr155 (d = 2.80–3.40 Å; force constant: 10 kcal mol^−1^ Å^−2^), between the oxygen of the OH-group in *meta*-position of **12** and the OD1 oxygen of the Asn152 side chain (d = 2.70–3.30 Å; force constant: 10 kcal mol^−1^ Å^−2^), and between the oxygen of the *para* OH-group of **12** and the NE2 nitrogen of the His221 side chain (d = 2.70–3.30 Å; force constant: 10 kcal mol^−1^ Å^−2^).

For the ternary complex of marmoset 17β-HSD1 with **12** and NADPH three additional distance restraints were used: between the keto-oxygen of **12** and the side chain oxygen of Ser142 (d = 2.50–3.10 Å; force constant: 10 kcal mol^−1^ Å^−2^), between the oxygen of the OH-group in *meta*-position of **1** and the OD1 oxygen of the Asn152 side chain (d = 2.40–3.00 Å; force constant: 10 kcal mol^−1^ Å^−2^), and between the oxygen of the *para* OH-group of **12** and the OD1 oxygen of the Asn282 side chain (d = 2.50–3.10 Å; force constant: 10 kcal mol^−1^ Å^−2^).

For the ternary complex of human 17β-HSD1 with **19** and NADPH three additional distance restraints were used: between the oxygen of the OH-group in 2-position of the naphthol core and the side chain oxygen of Tyr155 (d = 2.80–3.40 Å; force constant: 10 kcal mol^−1^ Å^−2^), between the oxygen of the OH-group in *meta*-position of the phenyl ring and the NE2 nitrogen of the His221 side chain (d = 3.00–4.20 Å; force constant: 10 kcal mol^−1^ Å^−2^), and between the nitrogen of the sulfonamide moiety of **19** and the OD1 oxygen of the Asn152 side chain (d = 2.70–3.40 Å; force constant: 10 kcal mol^−1^ Å^−2^).

For the ternary complex of marmoset 17β-HSD1 with **19** and NADPH two additional distance restraints were used: between the oxygen of the OH-group in 2-position of the naphthol core and the NE2 nitrogen of the His221 side chain (d = 2.70–3.40 Å; force constant: 10 kcal mol^−1^ Å^−2^) and between the oxygen of the OH-group in *meta*-position of the phenyl ring of **19** and the backbone carbonyl oxygen of Cys185 (d = 2.40–3.00 Å; force constant: 10 kcal mol^−1^ Å^−2^).

Trajectories were analyzed using the AMBER ptraj module, the MMTSB toolset [62] and the molecular visualization program VMD (Visual Molecular Dynamics) [63]. The resulting low energy structures were extracted for the homology model (apoform and ternary complex) and subjected to a subsequent minimization of 1000 steps (500 steps of steepest descent followed by 500 steps of conjugate gradient), using the sander module of AMBER. The modified generalized Born solvation model (IGB = 2) [64] was used. Active site volumes of low energy structures were calculated using the CASTp [45].

### Molecular docking

The three-dimensional structures used for docking studies were either retrieved from the PDB (1fdt, conformation B for residues 187–200) or from the homology modelling with subsequent MD simulation (equilibrated ternary complex after 5537 ps). The cocrystallized E2 and water molecules were removed from the PDB file. Hydrogen atoms and neutral end groups were added, NADP^+^ was turned into NADPH and correct atom types were set. Ionization states and hydrogen positions were assigned using the Protonate 3D utility of MOE2009.10 (Chemical Computing Group Inc., Montreal, Canada). Ligand structures were built in MOE and RESP charges were assigned as described above. The 17β-HSD1 three-dimensional structures and ligand structures were prepared for docking studies through the graphical user interface AutoDockTools4 [46]. For the ligands, non-polar hydrogen atoms were deleted, rotatable bonds were defined and RESP charges were kept. For the protein, non-polar hydrogen atoms were deleted and charges were added to the structure. Autodock4.2 [46] was used to dock the ligands in the steroidal binding site of the processed protein structures. A box, centered on the steroid-binding site, was set to define the docking area. Grid points of 90×90×90 with 0.250 Å spacing were calculated around the docking area for all the ligand atom types using AutoGrid4.2. For each inhibitor, 50 separate docking calculations were performed. Each docking calculation consisted of 25×10^5^ energy evaluations using the Lamarckian genetic algorithm local search (GALS) method. Each docking run was performed with a population size of 250. A mutation rate of 0.02 and a crossover rate of 0.8 were used to generate new docking trials for subsequent generations. The docking results from each of the 50 calculations were clustered on the basis of root-mean-square deviation (RMSD = 2.0 Å) between the Cartesian coordinates of the ligand atoms and were ranked on the basis of the free binding energy.

### Free energy calculations using the MM/PBSA method

The calculation of binding free energy was evaluated using the MM/PBSA (Molecular Mechanics/Poisson Boltzmann Surface Area) method as implemented in AMBER11 [65]. The electronic and Van der Waals energies were calculated using the sander module in AMBER11. The solvation free energy contributions may be further decomposed into an electrostatic and hydrophobic contribution. The electrostatic portion is calculated using the linearized PB equation. The hydrophobic contribution is approximated by the LCPO method [66] implemented within sander. The changes in entropy upon ligand association ΔS are estimated by normal mode analysis. For stable plateaus of the MD trajectories, snapshots were collected every 20^th^ frame (every 20 ps) and used to calculate relative binding affinity (ΔG_bind_) and absolute free energy (ΔG).

### Energy decomposition using the MM/GBSA method

A free energy decomposition of the protein ligand complexes was performed on a pairwise per-residue basis using the MM/GBSA (Molecular Mechanics/Generalized Born Surface Area) method as implemented in AMBER11. The GBSA implicit-solvent solvation model was used in order to avoid the retarding effect of the PBSA method.

### Inhibition assay

[2, 4, 6, 7-^3^H]-E1 and [2, 4, 6, 7-^3^H]-E2 were bought from Perkin Elmer, Boston. Quickszint Flow 302 scintillator fluid was bought from Zinsser Analytic, Frankfurt. Marmoset 17β-HSD1 and 17β-HSD2 were obtained from marmoset placenta according to previously described procedures [41]. Fresh marmoset placenta was homogenized and cytosolic and microsomal fractions were separated by centrifugation. For the partial purification of 17β-HSD1, the cytosolic fraction was precipitated with ammonium sulfate. 17β-HSD2 was obtained from the microsomal fraction.

#### Inhibition of 17β-HSD1

Inhibitory activities were evaluated by an established method with minor modifications [41]. Briefly, the enzyme preparation was incubated with NADH [500 µM] in the presence of potential inhibitors at 37°C in a phosphate buffer (50 mM) supplemented with 20% of glycerol and EDTA (1 mM). Inhibitor stock solutions were prepared in DMSO. The final concentration of DMSO was adjusted to 1% in all samples. The enzymatic reaction was started by addition of a mixture of unlabelled- and [2, 4, 6, 7-^3^H]-E1 (final concentration: 500 nM, 0.15 µCi). After 10 min, the reaction was stopped by the addition of HgCl_2_ (10 mM) and the mixture was extracted with diethylether. After evaporation, the steroids were dissolved in acetonitrile. E1 and E2 were separated using acetonitrile/water (45∶55) as mobile phase in a C18 reverse phase chromatography column (Nucleodur C18 Gravity, 3 µm, Macherey-Nagel, Düren) connected to an HPLC-system (Agilent 1200 Series, Agilent Technologies, Waldbronn). Detection and quantification of the steroids were performed using a radioflow detector (Agilent 1200 Series, Agilent Technologies, Waldbronn). The conversion rate was calculated after analysis of the resulting chromatograms according to the following equation: 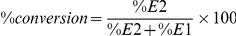. Each value was calculated from at least three independent experiments.

#### Inhibition of 17β-HSD2

The 17β-HSD2 inhibition assay was performed similarly to the 17β-HSD1 procedure. The microsomal fraction was incubated with NAD^+^ [1500 µM], test compound and a mixture of unlabelled- and [2, 4, 6, 7-^3^H]-E2 (final concentration: 500 nM, 0.11 µCi) for 20 min at 37°C. Further treatment of the samples and HPLC separation was carried out as mentioned above. The conversion rate was calculated after analysis of the resulting chromatograms according to the following equation: 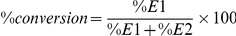.

## Supporting Information

Figure S1 **Energy profile drawn for the marmoset 17β-HSD1 model using PROSA.** Energy profiles of marmoset 17β-HSD1 in complex with NADPH (orange) and of marmoset 17β-HSD1 in complex with NADPH and E1 (green).
(TIF)

Figure S2 **Verify3D results for the marmoset 17β-HSD1 model.** Verify-3D results are shown for the secondary complex of marmoset 17β-HSD1 (orange) with NADPH and for ternary complex (green) with NADPH and E1; residues with positive score are reasonably folded.
(TIF)

Table S1 **Cluster analysis of molecular docking results.** All energies are expressed in kcal mol^−1^. The lowest energy conformation of each cluster, which is marked in bold was used for further investigation.
(DOC)

## References

[1] Poutanen M, Isomaa V, Peltoketo H, Vihko R (1995). Role of 17 beta-hydroxysteroid dehydrogenase type 1 in endocrine and intracrine estradiol biosynthesis.. J Steroid Biochem Mol Biol.

[2] Travis RC, Key TJ (2003). Oestrogen exposure and breast cancer risk.. Breast Cancer Res.

[3] Dizerega GS, Barber DL, Hodgen GD (1980). Endometriosis: role of ovarian steroids in initiation, maintenance and suppression.. Fertil Steril.

[4] Saloniemi T, Jarvensivu P, Koskimies P, Jokela H, Lamminen T (2010). Novel Hydroxysteroid (17β) Dehydrogenase 1 Inhibitors Reverse Estrogen-Induced Endometrial Hyperplasia in Transgenic Mice.. Am J Pathol.

[5] Gobbi S, Cavalli A, Rampa A, Belluti F, Piazzi L (2006). Lead optimization providing a series of flavone derivatives as potent nonsteroidal inhibitors of the cytochrome P450 aromatase enzyme.. J Med Chem.

[6] Cavalli A, Bisi A, Bertucci C, Rosini C, Paluszcak A (2005). Enantioselective nonsteroidal aromatase inhibitors identified through a multidisciplinary medicinal chemistry approach.. J Med Chem.

[7] Leonetti F, Favia A, Rao A, Aliano R, Paluszcak A (2004). Design, synthesis, and 3D QSAR of novel potent and selective aromatase inhibitors.. J Med Chem.

[8] Aggarwal S, Thareja S, Verma A, Bhardwaj T, Kumar M (2010). An overview on 5alpha-reductase inhibitors.. Steroids.

[9] Picard F, Schulz T, Hartmann RW (2002). 5-Phenyl substituted 1-methyl-2-pyridones and 4′-substituted biphenyl-4-carboxylic acids. synthesis and evaluation as inhibitors of steroid-5alpha-reductase type 1 and 2.. Bioorg Med Chem.

[10] Baston E, Hartmann RW (1999). N-substituted 4-(5-indolyl)benzoic acids. Synthesis and evaluation of steroid 5alpha-reductase type I and II inhibitory activity.. Bioorg Med Chem Lett.

[11] Baston E, Palusczak A, Hartmann RW (2000). 6-Substituted 1H-quinolin-2-ones and 2-methoxy-quinolines: synthesis and evaluation as inhibitors of steroid 5alpha reductases types 1 and 2.. Eur J Med Chem.

[12] Wetzel M, Marchais-Oberwinkler S, Hartmann RW (2011). 17β-HSD2 inhibitors for the treatment of osteoporosis: Identification of a promising scaffold.. Bioorg Med Chem.

[13] Jörnvall H, Persson B, Krook M, Atrian S, Gonzalez-Duarte R (1995). Short-chain dehydrogenases/reductases (SDR).. Biochemistry.

[14] Peltoketo H, Isomaa V, Mäentausta O, Vihko R (1988). Complete amino acid sequence of human placental 17 beta-hydroxysteroid dehydrogenase deduced from cDNA.. FEBS Lett.

[15] Ghosh D, Pletnev VZ, Zhu DW, Wawrzak Z, Duax WL (1995). Structure of human estrogenic 17beta- hydroxysteroid dehydrogenase at 2.20 Å resolution.. Structure.

[16] Filling C, Berndt KD, Benach J, Knapp S, Prozorovski T (2002). Critical residues for structure and catalysis in short-chain dehydrogenase/reductase.. J Biol Chem.

[17] Azzi A, Rehse PH, Zhu DW, Campbell RL, Labrie F (1996). Crystal structure of human estrogenic 17beta-hydroxysteroid dehydrogenase complexed with 17 beta-estradiol.. Nat Struct Biol.

[18] Marchais-Oberwinkler S, Henn C, Möller G, Klein T, Negri M (2010). 17β-Hydroxysteroid dehydrogenases (17β-HSDs) as therapeutic targets: Protein structures, functions and recent progress in inhibitor development.. J Steroid Biochem Mol Biol.

[19] Mazumdar M, Chen J, Lin SX Molecular basis of sex-steroid translation..

[20] Mazumdar M, Chen J, Lin SX Molecular basis of sex-steroids: thier translational activity..

[21] Negri M, Recanatini M, Hartmann RW (2010). Insights in 17β-HSD1 Enzyme Kinetics and Ligand Binding by Dynamic Motion Investigation.. PLoS ONE.

[22] Brožic P, Lanišnik Rižner T, Gobec S (2008). Inhibitors of 17beta-hydroxysteroid dehydrogenase type 1.. Curr Med Chem.

[23] Poirier D (2009). Advances in development of inhibitors of 17beta hydroxysteroid dehydrogenases.. Anticancer Agents Med Chem.

[24] Day JM, Tutill HJ, Purohit A (2010). 17β-Hydroxysteroid dehydrogenase inhibitors.. Minerva Endocrinol.

[25] Day JM, Tutill HJ, Purohit A, Reed MJ (2008). Design and validation of specific inhibitors of 17beta-hydroxysteroid dehydrogenases for therapeutic application in breast and prostate cancer, and in endometriosis.. Endocr Relat Cancer.

[26] Messinger J, Hirvelä L, Husen B, Kangas L, Koskimies P (2006). New inhibitors of 17beta-hydroxysteroid dehydrogenase type 1.. Mol Cell Endocrinol.

[27] Karkola S, Lilienkampf A, Wähälä K (2008). A 3D QSAR model of 17beta-HSD1 inhibitors based on a thieno[2,3-d]pyrimidin-4(3H)-one core applying molecular dynamics simulations and ligand-protein docking.. ChemMedChem.

[28] Frotscher M, Ziegler E, Marchais-Oberwinkler S, Kruchten P, Neugebauer A (2008). Design, synthesis, and biological evaluation of (hydroxyphenyl)naphthalene and -quinoline derivatives: potent and selective nonsteroidal inhibitors of 17β-hydroxysteroid dehydrogenase type 1 (17β-HSD1) for the treatment of estrogen-dependent diseases.. J Med Chem.

[29] Marchais-Oberwinkler S, Frotscher M, Ziegler E, Werth R, Kruchten P (2009). Structure-activity study in the class of 6-(3′-hydroxyphenyl)naphthalenes leading to an optimization of a pharmacophore model for 17β-hydroxysteroid dehydrogenase type 1 (17β-HSD1) inhibitors.. Mol Cell Endocrinol.

[30] Marchais-Oberwinkler S, Kruchten P, Frotscher M, Ziegler E, Neugebauer A (2008). Substituted 6-phenyl-2-naphthols. Potent and selective nonsteroidal inhibitors of 17β-hydroxysteroid dehydrogenase type 1 (17β-HSD1): design, synthesis, biological evaluation, and pharmacokinetics.. J Med Chem.

[31] Marchais-Oberwinkler S, Wetzel M, Ziegler E, Kruchten P, Werth R (2011). New Drug-Like Hydroxyphenylnaphthol Steroidomimetics As Potent and Selective 17β-Hydroxysteroid Dehydrogenase Type 1 Inhibitors for the Treatment of Estrogen-Dependent Diseases.. J Med Chem.

[32] Bey E, Marchais-Oberwinkler S, Kruchten P, Frotscher M, Werth R (2008). Design, synthesis and biological evaluation of bis (hydroxyphenyl) azoles as potent and selective non-steroidal inhibitors of 17β-hydroxysteroid dehydrogenase type 1 (17β-HSD1) for the treatment of estrogen-dependent diseases.. Bioorg Med Chem.

[33] Bey E, Marchais-Oberwinkler S, Negri M, Kruchten P, Oster A (2009). New Insights into the SAR and Binding Modes of Bis(hydroxyphenyl)thiophenes and -benzenes: Influence of Additional Substituents on 17β-Hydroxysteroid Dehydrogenase Type 1 (17β-HSD1) Inhibitory Activity and Selectivity.. J Med Chem.

[34] Bey E, Marchais-Oberwinkler S, Werth R, Negri M, Al-Soud YA (2008). Design, synthesis, biological evaluation and pharmacokinetics of bis(hydroxyphenyl) substituted azoles, thiophenes, benzenes, and aza-benzenes as potent and selective nonsteroidal inhibitors of 17β-hydroxysteroid dehydrogenase type 1 (17β-HSD1).. J Med Chem.

[35] Oster A, Hinsberger S, Werth R, Marchais-Oberwinkler S, Frotscher M (2010). Bicyclic Substituted Hydroxyphenylmethanones as Novel Inhibitors of 17β-Hydroxysteroid Dehydrogenase Type 1 (17β-HSD1) for the Treatment of Estrogen-Dependent Diseases.. J Med Chem.

[36] Oster A, Klein T, Henn C, Werth R, Marchais-Oberwinkler S (2011). Bicyclic Substituted Hydroxyphenylmethanone Type Inhibitors of 17β-Hydroxysteroid Dehydrogenase Type 1 (17β-HSD1): The Role of the Bicyclic Moiety.. ChemMedChem.

[37] Oster A, Klein T, Werth R, Kruchten P, Bey E (2010). Novel estrone mimetics with high 17β-HSD1 inhibitory activity.. Bioorg Med Chem.

[38] Negri M, Recanatini M, Hartmann RW (2011). Computational investigation of the binding mode of bis(hydroxylphenyl)arenes in 17β-HSD1: molecular dynamics simulations, free energy calculations, and molecular electrostatic potential maps.. J Comput Aided Mol Des.

[39] Möller G, Husen B, Kowalik D, Hirvelä L, Plewczynski D (2010). Species Used for Drug Testing Reveal Different Inhibition Susceptibility for 17beta-Hydroxysteroid Dehydrogenase Type 1.. PLoS ONE.

[40] Kruchten P, Werth R, Marchais-Oberwinkler S, Bey E, Ziegler E (2009). Development of biological assays for the identification of selective inhibitors of estradiol formation from estrone in rat liver preparations.. C R Chim.

[41] Kruchten P, Werth R, Marchais-Oberwinkler S, Frotscher M, Hartmann RW (2009). Development of a biological screening system for the evaluation of highly active and selective 17β-HSD1-inhibitors as potential therapeutic agents.. Mol Cell Endocrinol.

[42] Sali A, Blundell TL (1993). Comparative protein modelling by satisfaction of spatial restraints.. J Mol Biol.

[43] Shen M-Y, Sali A (2006). Statistical potential for assessment and prediction of protein structures.. Protein Sci.

[44] Laskowski RA, MacArthur MW, Moss DS, Thornton JM (1993). PROCHECK: a program to check the stereochemical quality of protein structures.. J App Cryst.

[45] Dundas J, Ouyang Z, Tseng J, Binkowski A, Turpaz Y (2006). CASTp: computed atlas of surface topography of proteins with structural and topographical mapping of functionally annotated residues.. Nucleic Acids Res.

[46] Morris GM, Huey R, Lindstrom W, Sanner MF, Belew RK (2009). AutoDock4 and AutoDockTools4: Automated Docking with Selective Receptor Flexibility.. J Comput Chem.

[47] Richardson JS, Richardson DC (1988). Amino acid preferences for specific locations at the ends of alpha helices.. Science.

[48] Kumar S, Bansal M (1998). Dissecting alpha-helices: position-specific analysis of alpha-helices in globular proteins.. Proteins.

[49] Lilienkampf A, Karkola S, Alho-Richmond S, Koskimies P, Johansson N (2009). Synthesis and biological evaluation of 17β-hydroxysteroid dehydrogenase type 1 (17β-HSD1) inhibitors based on a thieno[2,3-d]pyrimidin-4(3H)-one core.. J Med Chem.

[50] Messinger J, Husen B, Koskimies P, Hirvelä L, Kallio L (2009). Estrone C15 derivatives–a new class of 17β-hydroxysteroid dehydrogenase type 1 inhibitors.. Mol Cell Endocrinol.

[51] Katoh K, Kuma K, Toh H, Miyata T (2005). MAFFT version 5: improvement in accuracy of multiple sequence alignment.. Nucleic Acids Res.

[52] Sippl MJ (1993). Recognition of errors in three-dimensional structures of proteins.. Proteins.

[53] Colovos C, Yeates TO (1993). Verification of protein structures: patterns of nonbonded atomic interactions.. Protein Sci.

[54] Lüthy R, Bowie JU, Eisenberg D (1992). Assessment of protein models with three-dimensional profiles.. Nature.

[55] Case D, Darden T, Cheatham T, Simmerling C (2006). AMBER 9..

[56] Schmidt M, Baldridge K, Boatz J, Elbert S, Gordon M (1993). General atomic and molecular electronic structure system.. J Comput Chem.

[57] Bayly CI, Cieplak P, Cornell WD, Kollman PA (1993). A well behaved electrostatic potential based method using charge restraints for determining atom-centered charges: the RESP model.. J Phys Chem.

[58] Hornak V, Abel R, Okur A, Strockbine B, Roitberg A (2006). Comparison of multiple Amber force fields and development of improved protein backbone parameters.. Proteins.

[59] Wang J, Wolf RM, Caldwell JW, Kollman PA, Case DA (2004). Development and testing of a general amber force field.. J Comput Chem.

[60] Darden T, Perera L, Li L, Pedersen L (1999). New tricks for modelers from the crystallography toolkit: the particle mesh Ewald algorithm and its use in nucleic acid simulations.. Structure.

[61] Ryckaert J-P, Ciccotti G, Berendsen HJC (1977). Numerical integration of the cartesian equations of motion of a system with constraints: molecular dynamics of n-alkanes.. J Comput Phys.

[62] Feig M, Karanicolas J, Brooks CL (2004). MMTSB Tool Set: enhanced sampling and multiscale modeling methods for applications in structural biology.. J Mol Graph Model.

[63] Humphrey W, Dalke A, Schulten K (1996). VMD: visual molecular dynamics.. J Mol Graph.

[64] Onufriev A, Bashford D, Case DA (2004). Exploring protein native states and large-scale conformational changes with a modified generalized born model.. Proteins.

[65] Kollman PA, Massova I, Reyes C, Kuhn B, Huo S (2000). Calculating structures and free energies of complex molecules: combining molecular mechanics and continuum models.. Acc Chem Res.

[66] Weiser J, Shenkin PS, Still WC (1999). Approximate solvent-accessible surface areas from tetrahedrally directed neighbor densities.. Biopolymers.

