# Supplementary figures and images for: Structural Basis for Species Specific Inhibition of 17β-Hydroxysteroid Dehydrogenase Type 1 (17β-HSD1): Computational Study and Biological Validation
Source: PLoS One. 2011 Aug 9;6(8):e22990. doi: 10.1371/journal.pone.0022990 (PMC3153478; doi:10.1371/journal.pone.0022990)

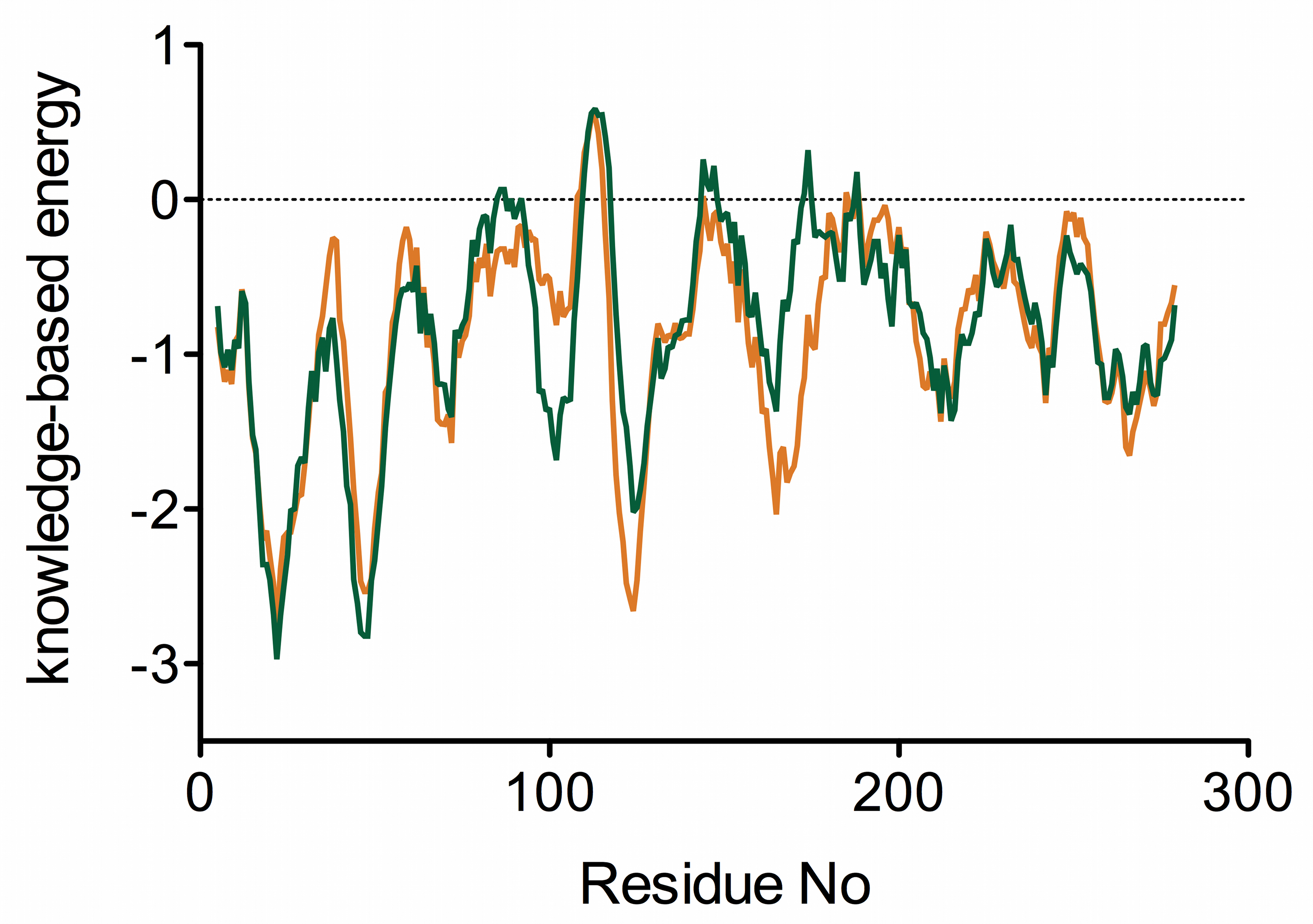

Supplement: Figure S1 — Energy profile drawn for the marmoset 17β-HSD1 model using PROSA. Energy profiles of marmoset 17β-HSD1 in complex with NADPH (orange) and of marmoset 17β-HSD1 in complex with NADPH and E1 (green). (TIF) [file pone.0022990.s001.tif]

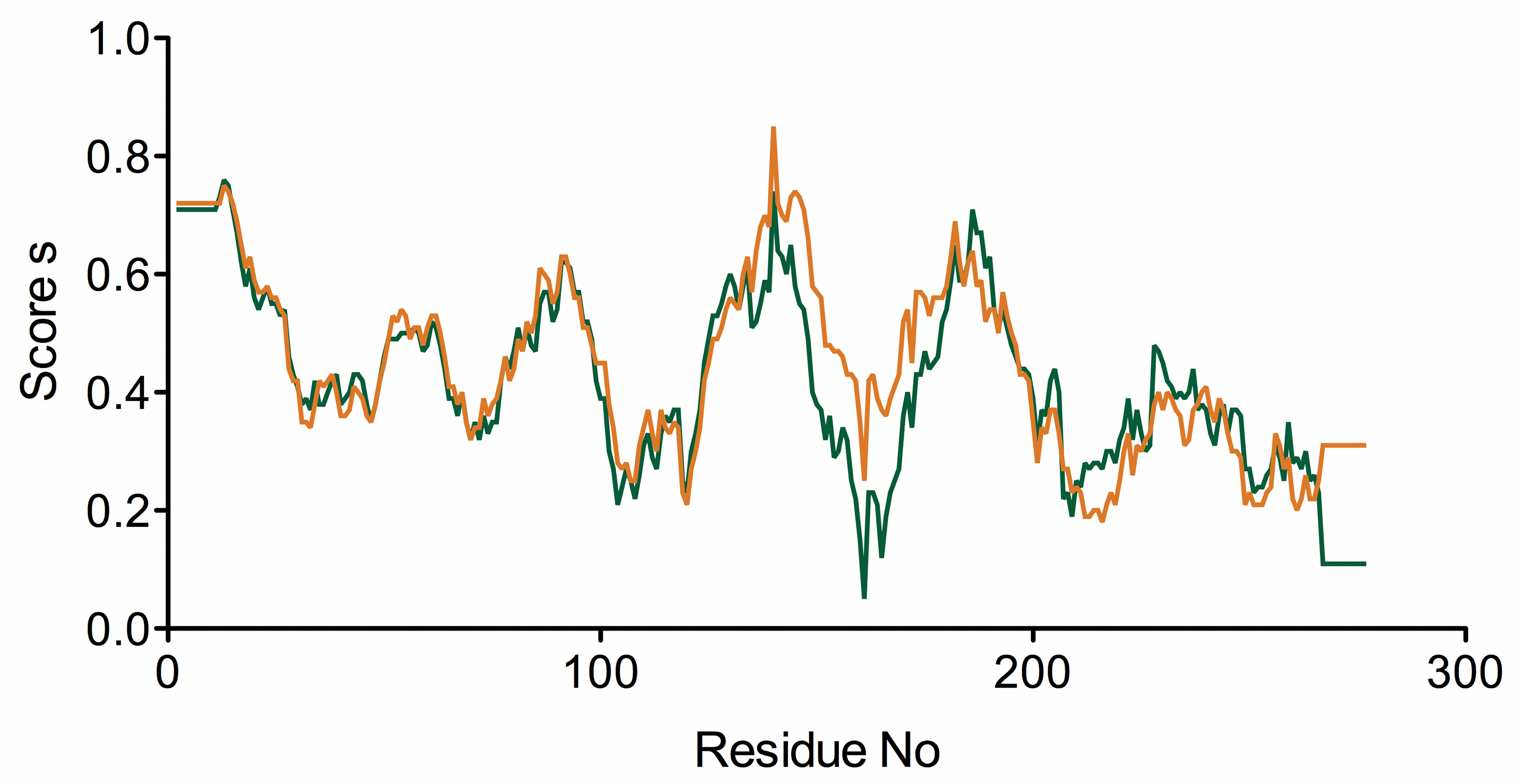

Supplement: Figure S2 — Verify3D results for the marmoset 17β-HSD1 model. Verify-3D results are shown for the secondary complex of marmoset 17β-HSD1 (orange) with NADPH and for ternary complex (green) with NADPH and E1; residues with positive score are reasonably folded. (TIF) [file pone.0022990.s002.tif]
