# Supplementary material for: Structural Basis for Species Specific Inhibition of 17β-Hydroxysteroid Dehydrogenase Type 1 (17β-HSD1): Computational Study and Biological Validation
Source: PLoS One. 2011 Aug 9;6(8):e22990. doi: 10.1371/journal.pone.0022990 (PMC3153478; doi:10.1371/journal.pone.0022990)
Supplement: Table S1 — Cluster analysis of molecular docking results. All energies are expressed in kcal mol−1. The lowest energy conformation of each cluster, which is marked in bold was used for further investigation. (DOC) [file pone.0022990.s003.doc]

| **Comp** | **No of clusters** | **Cluster** | **No of conformations** | **Best binding energy (∆G_bind_)** |
| --- | --- | --- | --- | --- |
| **Human x-ray structure (1fdtB)** | | | |  |
| **12** | 3 | 1 | 2 | -4.77 |
|  |  | **2** | 45 | -4.65 |
|  |  | 3 | 3 | -4.58 |
| **19** | 5 | **1** | 24 | -5.94 |
|  |  | 2 | 1 | -5.44 |
|  |  | 3 | 19 | -4.98 |
|  |  | 4 | 1 | -4.92 |
|  |  | 5 | 5 | -4.87 |
| **Marmoset monkey homology model** | | | |  |
| **12** | 3 | **1** | 21 | -5.60 |
|  |  | 2 | 27 | -5.41 |
|  |  | 3 | 1 | -5.0 |
|  |  | 4 | 1 | -4.59 |
| **19** | 5 | 1 | 7 | -5.79 |
|  |  | **2** | 33 | -5.74 |
|  |  | 3 | 3 | -5.11 |
|  |  | 4 | 5 | -4.41 |
|  |  | 5 | 2 | -4.10 |
